# Atopic Dermatitis Beyond the Skin Barrier: Precision Medicine Approaches to Immunological Profiling and Therapeutic Innovation

**DOI:** 10.3390/ijms27146129

**Published:** 2026-07-09

**Authors:** Virgilios Galatis, Isabela Siloși, Mohamed-Zakaria Assani, Lidia Boldeanu, George G. Mitroi, Mihail Virgil Boldeanu

**Affiliations:** 1 Doctoral School, University of Medicine and Pharmacy of Craiova, 200349 Craiova, Romania; virgil.galatis@gmail.com (V.G.); mohamed.assani@umfcv.ro (M.-Z.A.); 2 Department of Immunology, Faculty of Medicine, University of Medicine and Pharmacy of Craiova, 200349 Craiova, Romania; isabela_silosi@yahoo.com (I.S.); mihail.boldeanu@umfcv.ro (M.V.B.); 3 Department of Microbiology, Faculty of Medicine, University of Medicine and Pharmacy of Craiova, 200349 Craiova, Romania; 4 Department of Dermatology, University of Medicine and Pharmacy of Craiova, 200349 Craiova, Romania

**Keywords:** atopic dermatitis, precision medicine, cytokines, biomarkers, Th2 inflammation, biologics, JAK inhibitors, immunopathogenesis

## Abstract

Atopic dermatitis (AD) is a chronic, relapsing inflammatory skin disease characterized by substantial clinical and immunological heterogeneity. Once considered primarily a disorder of epidermal barrier dysfunction, AD is now recognized as a complex systemic inflammatory condition involving dysregulated immune responses, epithelial-derived signaling, neuroimmune interactions, and diverse molecular endotypes. Advances in molecular immunology have substantially improved understanding of the cytokine networks underlying disease pathogenesis and have accelerated the transition toward precision medicine approaches in AD. This narrative review summarizes current evidence regarding the immunopathogenesis of AD, focusing on the interplay between classical and emerging cytokine pathways, biomarker development, and recent therapeutic innovations. While interleukin (IL)-4 and IL-13 remain central drivers of type 2 inflammation and barrier impairment, additional mediators including IL-31, IL-33, IL-22, thymic stromal lymphopoietin (TSLP), and OX40/OX40L signaling, and the emerging Th9/IL-9 axis contribute to chronic inflammation, neuroimmune activation, epidermal remodeling, pruritus, and disease heterogeneity. Comparative evaluation of these pathways supports the identification of distinct immunological endotypes relevant to disease stratification and targeted therapy. The review further discusses current and emerging biomarkers associated with disease severity, therapeutic responsiveness, and inflammatory profiling, including cytokine signatures, serum biomarkers, and transcriptomic approaches. Recent advances in biologic therapies, Janus kinase (JAK) inhibitors, and novel cytokine-targeted interventions are discussed within the context of a precision medicine framework integrating immunological profiling, molecular endotyping, and mechanism-based therapeutic innovation. Continued advances in biomarker discovery, multi-omics technologies, and predictive therapeutic algorithms are expected to further refine disease stratification and support increasingly individualized management strategies for patients with AD.

## 1. Introduction

Atopic dermatitis (AD) has evolved from being considered primarily an epidermal barrier disorder to a highly heterogeneous systemic inflammatory disease characterized by complex immune dysregulation, diverse molecular endotypes, and variable therapeutic responses [1,2]. As one of the most common chronic inflammatory skin diseases worldwide, AD affects both pediatric and adult populations and represents a major public health burden due to its chronic relapsing course, intense pruritus, sleep disturbance, psychosocial impact, and reduced quality of life [1,3].

Although epidermal barrier dysfunction remains a central component of AD pathogenesis, growing evidence demonstrates that the disease extends far beyond structural skin abnormalities [1,4]. Contemporary models emphasize dynamic interactions between keratinocyte dysfunction, type 2 immune activation, neuroimmune signaling, environmental triggers, and systemic inflammatory pathways. This evolving understanding has accelerated the development of precision medicine strategies to identify individualized disease mechanisms and therapeutic targets [4,5].

The immunological landscape of AD is predominantly driven by T helper 2 (Th2)-mediated inflammation, with interleukin (IL)-4 and IL-13 playing key roles in epidermal barrier impairment, immunoglobulin E (IgE) production, eosinophilic inflammation, and chronic itch [1,2]. However, recent studies have demonstrated that additional immune pathways, including Th17-, Th22-, and Th1-associated responses, contribute significantly to disease chronicity, lesion heterogeneity, and ethnic or age-related phenotypes [1,4]. Consequently, AD is increasingly recognized as a spectrum of immunological endotypes rather than a single disease entity [3,5].

Within this evolving framework, cytokine profiling has emerged as a promising strategy for improving disease stratification and therapeutic personalization [3,5]. While classical type 2 cytokines remain central to AD immunopathogenesis, increasing evidence indicates that additional cytokine pathways contribute to disease heterogeneity, chronic inflammation, neuroimmune interactions, and tissue remodeling [1,6]. These advances have stimulated growing interest in cytokine-based biomarkers that can define clinically relevant endotypes, predict therapeutic response, and support precision medicine approaches in AD. The individual cytokine pathways and their biological and clinical significance are discussed in the following sections.

The transition toward precision medicine has been further accelerated by major therapeutic advances in moderate-to-severe AD [5,7]. The introduction of biologic agents targeting IL-4/IL-13 signaling pathways, alongside Janus kinase inhibitors (JAKi) that modulate multiple cytokine cascades, has revolutionized treatment strategies and significantly improved clinical outcomes [7,8]. Nevertheless, considerable interindividual variability in treatment response persists, highlighting the need for reliable immunological profiling tools and biomarker-guided therapeutic algorithms [3,5].

In this context, precision medicine aims to integrate clinical phenotypes with molecular and immunological signatures to optimize individualized therapeutic strategies [4,5]. The biological basis of these approaches, including the contribution of established and emerging cytokine pathways, is discussed in the following sections.

Therefore, this narrative review provides an updated overview of the immunopathogenesis of AD, emphasizing the interplay between cytokine networks, immunological profiling, biomarker development, and targeted therapeutic innovations. The following sections discuss how these advances support precision medicine approaches and may contribute to improved disease stratification and individualized patient management.

## 2. Immunopathogenesis of Atopic Dermatitis: Beyond the Skin Barrier

Building on the concepts introduced above, current research has focused on elucidating the cellular and molecular mechanisms underlying disease heterogeneity in AD. These mechanisms involve dynamic interactions among epidermal barrier dysfunction, epithelial-derived cytokines, innate and adaptive immune responses, neuroimmune pathways, and environmental factors, collectively shaping distinct inflammatory endotypes and therapeutic responsiveness [1,2,3,4,5].

### 2.1. Epidermal Barrier Dysfunction and the Active Role of Keratinocytes

Epidermal barrier impairment remains one of the earliest pathogenic events in AD and represents a critical interface between environmental stimuli and immune activation [1,2]. The healthy epidermis functions as both a physical and an immunological barrier that protects against allergens, microbial antigens, and pollutants, and helps prevent transepidermal water loss. In AD, disruption of this protective function facilitates increased penetration of environmental triggers, thereby initiating and perpetuating chronic cutaneous inflammation.

Several structural abnormalities contribute to epidermal barrier dysfunction in AD, including reduced filaggrin expression, altered ceramide composition, impaired tight junction integrity, and defective keratinocyte differentiation [1,4]. Although filaggrin deficiency has been strongly associated with impaired barrier integrity, contemporary evidence indicates that barrier dysfunction in AD cannot be explained solely through structural protein abnormalities. Instead, inflammatory cytokines themselves actively contribute to epidermal damage by suppressing the expression of proteins essential for epidermal differentiation and barrier maintenance.

Among the major cytokines involved in this process, IL-4 and IL-13 exert profound inhibitory effects on the synthesis of filaggrin, loricrin, and involucrin, thereby perpetuating epidermal fragility and increasing susceptibility to environmental insults [1,4]. This reciprocal relationship between inflammation and barrier dysfunction establishes a self-amplifying pathogenic cycle in which epidermal disruption promotes immune activation, while inflammatory mediators further aggravate barrier impairment.

Keratinocytes are now recognized as active immunological participants rather than passive structural cells. Following epidermal injury or exposure to allergens and irritants, keratinocytes release epithelial-derived cytokines (alarmins), including TSLP, IL-25, and IL-33, which initiate and amplify type 2 inflammatory responses by activating dendritic cells, innate lymphoid cells, eosinophils, mast cells, and Th2 lymphocytes [4,6].

Among epithelial alarmins, TSLP and IL-33 have emerged as key mediators of AD pathogenesis. Increased TSLP expression has been demonstrated in lesional AD skin and correlates with disease activity [9]. Beyond its role in initiating type 2 immune responses, TSLP contributes to chronic immune polarization and has become an attractive therapeutic target [4,9]. IL-33 also plays a central role in amplifying epithelial-driven inflammation and has been implicated in pruritus signaling, chronic tissue remodeling, and sustained disease activity, further supporting its potential as a therapeutic target [6,10].

Collectively, these findings establish the epidermis as an active immunological organ that not only provides structural protection but also orchestrates inflammatory responses through epithelial–immune crosstalk, thereby sustaining chronic disease activity.

### 2.2. Type 2 Inflammation as the Central Immunological Axis

Type 2 immune activation is the dominant immunological hallmark of AD and is primarily mediated by interactions among Th2 lymphocytes, ILC2s, dendritic cells, eosinophils, mast cells, and keratinocytes [1,2]. Cytokines, including IL-4, IL-13, and IL-5, orchestrate most acute inflammatory responses observed in AD and directly contribute to epidermal barrier disruption, eosinophilic inflammation, IgE synthesis, and chronic pruritus.

IL-4 and IL-13 are considered the principal drivers of AD immunopathogenesis and have become central therapeutic targets in modern biologic therapy [7,8]. These cytokines signal through shared receptor pathways and activate JAK and signal transducer and activator of transcription (JAK-STAT) signaling, thereby amplifying type 2 immune responses and suppressing antimicrobial defense mechanisms [11]. Their detrimental effects on epidermal barrier integrity, described in the previous section, further reinforce the self-perpetuating interaction between barrier dysfunction and chronic inflammation [1,4].

Although IL-4 and IL-13 exhibit overlapping biological functions, increasing evidence suggests that they may contribute differently to disease pathogenesis. IL-4 appears particularly important in early stages of Th2 polarization, whereas IL-13 is more closely associated with chronic tissue inflammation, epidermal remodeling, and sustained disease activity [4,11]. Consistently, increased IL-13 expression in lesional skin has been strongly correlated with disease severity [12].

IL-5 also contributes significantly to eosinophilic inflammation by activating, recruiting, and sustaining eosinophils within cutaneous tissues [1]. Elevated eosinophil counts frequently correlate with severe disease phenotypes and may reflect systemic type 2 inflammatory activation.

In addition to adaptive immunity, innate immune mechanisms amplify type 2 inflammation through the activity of ILC2s, which produce IL-5 and IL-13 independently of antigen-specific adaptive immune responses [6,8]. Together with epithelial-derived alarmins discussed in Section 2.1, these cells contribute to the initiation and maintenance of early inflammatory responses in AD.

The pathogenic importance of type 2 inflammation is reflected by the remarkable efficacy of biologic agents targeting IL-4 and IL-13 signaling pathways, including dupilumab, tralokinumab, and lebrikizumab (see Section 6 for a detailed discussion) [7,8].

### 2.3. Beyond Th2 Dominance: Additional Inflammatory Pathways and Disease Heterogeneity

Although type 2 inflammation remains the dominant immunological axis in AD, recent studies have demonstrated that the disease involves substantially more complex inflammatory networks than previously recognized [1,4]. Chronic AD lesions often exhibit simultaneous activation of Th1-, Th17-, and Th22-associated pathways, contributing to disease chronicity, epidermal hyperplasia, treatment resistance, and phenotypic diversity.

Th22 cells and their signature cytokine IL-22 play a central role in epidermal remodeling and lichenification by promoting keratinocyte proliferation and impairing terminal differentiation [6,13]. Elevated IL-22 expression has consistently been associated with chronic AD and may represent a useful biomarker of disease progression.

Similarly, Th17-related cytokines appear to contribute to specific AD phenotypes, particularly pediatric and Asian variants characterized by enhanced IL-17 signaling and neutrophilic inflammation [4]. IL-17 may additionally influence antimicrobial peptide expression and contribute to microbial dysregulation within the epidermal microenvironment.

Chronic lesions also exhibit increased expression of interferon-γ (IFN-γ), reflecting progressive Th1 activation during the prolonged evolution of the disease [1]. This transition toward mixed inflammatory profiles may partially explain why some patients exhibit incomplete responses to therapies targeting isolated type 2 pathways.

Collectively, these findings support the concept that distinct inflammatory endotypes underlie the clinical heterogeneity of AD and may account for differences in disease severity, phenotype, and therapeutic responsiveness [3,5].

Recent molecular profiling studies have identified significant differences among intrinsic and extrinsic AD, pediatric and adult disease, and acute versus chronic lesions, further supporting biomarker-based patient stratification and individualized therapeutic approaches [3,14].

### 2.4. Neuroimmune Interactions and the Molecular Basis of Pruritus

Pruritus represents the hallmark symptom of AD and is increasingly recognized as a consequence of highly complex neuroimmune interactions involving cytokines, epidermal inflammation, peripheral sensory neurons, and central nervous system signaling pathways [6]. Chronic itch significantly contributes to sleep disturbance, psychological stress, impaired quality of life, and perpetuation of the itch-scratch cycle.

Among the emerging cytokines implicated in AD, IL-31 has attracted particular attention because of its central role in neuroimmune signaling and chronic pruritus [6,10]. The biological functions and clinical relevance of IL-31 are discussed in greater detail in Section 3.3.

IL-31 promotes neuronal hypersensitivity and chronic itch, thereby sustaining the itch–scratch cycle and amplifying cutaneous inflammation [6,10]. Elevated serum IL-31 concentrations have consistently been associated with pruritus severity, sleep impairment, and increased disease activity, supporting its value as both a pathogenic mediator and a potential biomarker.

Neuroimmune signaling in AD also involves epithelial-derived cytokines, particularly TSLP and IL-33, which further amplify neuronal sensitization and contribute to persistent pruritus and chronic inflammation [6].

The recognition of neuroimmune pathways as central pathogenic mechanisms has generated significant therapeutic interest in cytokines associated with pruritus signaling. Nemolizumab, an anti-IL-31 receptor monoclonal antibody, exemplifies this translational approach, whereas its clinical efficacy and safety profile are discussed in detail in Section 6 [15].

### 2.5. Immunological Endotypes and Precision Medicine Perspectives

The marked immunological heterogeneity of AD has shifted clinical research toward precision medicine strategies based on immunological profiling and disease endotyping [3,5]. Integrating clinical phenotypes with molecular characteristics may improve patient stratification and therapeutic selection.

Recent advances in transcriptomic and immunological profiling have demonstrated that cytokine expression patterns vary significantly according to age, ethnicity, disease chronicity, and treatment exposure [5,14]. These findings reinforce the concept that AD comprises multiple immunological endotypes and highlight the potential value of molecular signatures for predicting disease behavior and therapeutic response.

Consequently, characterization of inflammatory endotypes has become a major objective of contemporary AD research. The biomarkers currently under investigation and their potential clinical applications are discussed in Section 4.

## 3. Cytokine Networks and Emerging Immunological Targets

Building upon the immunopathogenic mechanisms described in Section 2, AD is now recognized as a disease driven by interconnected cytokine networks that influence epidermal barrier integrity, neuroimmune signaling, tissue remodeling, and therapeutic responsiveness [1,4,10,13,16]. Molecular profiling studies have further demonstrated that these cytokine signatures vary according to disease stage, age, ethnicity, severity, and treatment exposure, contributing to the marked immunological heterogeneity of AD [3,5].

Although type 2 cytokines remain central to AD pathogenesis, increasing evidence indicates that additional inflammatory mediators contribute to disease chronicity, epidermal remodeling, neuroimmune activation, and clinical heterogeneity [6,10,16]. The following sections compare the biological and clinical significance of these classical and emerging cytokine pathways.

Rather than acting as isolated mediators, cytokines function within interconnected inflammatory circuits that shape disease endotypes and influence therapeutic responsiveness. Understanding these interactions provides the basis for the biomarker and therapeutic perspectives discussed in the following sections.

### 3.1. Classical Th2 Cytokines: IL-4, IL-13, and IL-5

Type 2 inflammation remains the central immunological hallmark of AD and is largely mediated through IL-4, IL-13, and IL-5 signaling pathways [1,2].

As discussed in Section 2.2, these cytokines play central roles in immune polarization, epidermal barrier dysfunction, eosinophilic inflammation, and chronic disease progression. Among them, IL-4 and IL-13 have emerged as the most clinically relevant mediators due to their strong association with disease severity and their successful therapeutic targeting [7,8,12]. Beyond their pathogenic roles, IL-4- and IL-13-related molecular signatures are increasingly investigated as biomarkers supporting disease stratification and treatment response [3,5]. Elevated IL-13 expression, in particular, has consistently been associated with lesional skin inflammation and more severe disease phenotypes, supporting its potential value in patient stratification and therapeutic selection [12]. The clinical relevance of the IL-4/IL-13 axis is reflected by the efficacy of biologic therapies targeting these pathways, including dupilumab, tralokinumab, and lebrikizumab (see Section 6 for detailed discussion) [7,8].

IL-5 remains an important contributor to eosinophilic inflammation and may provide complementary information regarding systemic type 2 immune activation. Although IL-5-directed therapies have shown limited efficacy in AD, eosinophil-associated pathways remain relevant biomarkers of disease activity and inflammatory burden [1].

Collectively, IL-4, IL-13, and IL-5 define the core type 2 inflammatory endotype and provide the biological basis for cytokine-guided patient stratification in AD.

### 3.2. Epithelial Alarmins and Early Immune Activation

Increasing evidence indicates that epithelial-derived cytokines, commonly referred to as alarmins, play a central role in initiating and amplifying inflammatory responses in AD [4,6]. Among these mediators, TSLP, IL-25, and IL-33 have emerged as key regulators of early immune activation and epithelial-immune communication.

As outlined in Section 2.1, TSLP is a key epithelial alarmin linking epidermal barrier disruption with type 2 immune activation. Elevated TSLP expression in lesional skin correlates with disease activity and highlights its potential as both a biomarker and a therapeutic target [4,9].

Beyond its role in adaptive immunity, TSLP contributes to neuroimmune activation and may directly influence pruritus through interactions with peripheral sensory neurons [8,10]. These additional functions further support its relevance as a therapeutic target in AD.

Similar to TSLP, IL-33 amplifies epithelial-driven type 2 inflammation and has been implicated in severe AD phenotypes, enhanced eosinophilic inflammation, chronic pruritus, and tissue remodeling [6,10]. Its pleiotropic biological effects further support IL-33 as a promising biomarker and emerging therapeutic target.

IL-25 also contributes to Th2 polarization by activating ILC2s and enhancing type 2 cytokine production [4]. Although less extensively studied than TSLP and IL-33, IL-25 is increasingly recognized as an important mediator of epithelial-driven inflammation in AD.

The therapeutic potential of epithelial alarmins is discussed in greater detail in Section 6. Briefly, biologic agents targeting TSLP and IL-33 are currently under clinical investigation and may represent future treatment options for patients with severe or treatment-resistant AD [7].

### 3.3. IL-31 and the Neuroimmune Axis

Building upon the neuroimmune mechanisms described in Section 2.4, IL-31 has emerged as a promising biomarker and therapeutic target in AD. Elevated serum IL-31 levels have been consistently associated with increased disease severity, chronic pruritus, sleep disturbance, and impaired quality of life, supporting its potential utility as a marker of neuroimmune activation and disease burden [6,10,13]. The clinical relevance of IL-31 extends beyond symptom generation. Recent evidence suggests that enhanced IL-31 signaling may contribute to epidermal barrier dysfunction, chronic inflammatory amplification, and persistence of the itch-scratch cycle, thereby linking neuroimmune dysregulation with disease chronicity [6,13]. Consequently, IL-31 has become one of the most extensively investigated cytokines for biomarker-guided therapeutic stratification in AD.

The therapeutic relevance of IL-31 is illustrated by nemolizumab, a monoclonal antibody targeting the IL-31 receptor α [15,16]. Clinical studies have demonstrated significant improvements in pruritus, sleep quality, and overall disease control, whereas its broader clinical implications, including safety considerations and cutaneous adverse events, are discussed in Section 6 [15,16]. These findings establish IL-31 as both a biomarker of neuroimmune activation and a clinically actionable therapeutic target.

### 3.4. Th22 Responses, IL-22, and Epidermal Remodeling

As discussed in Section 2.3, IL-22 is a key mediator of epidermal remodeling, lichenification, and chronic tissue inflammation in AD [13]. Beyond its role in promoting keratinocyte proliferation and impaired epidermal differentiation, IL-22 has been consistently associated with chronic lesions, epidermal hyperplasia, and lichenification. These findings support its value as a biomarker of chronic disease activity and a potential therapeutic target, particularly in patients with longstanding or treatment-resistant AD [13].

Recent molecular profiling studies have demonstrated that IL-22 expression varies according to ethnicity and disease phenotype, with particularly strong activation reported in Asian AD populations [14,17,18,19]. These observations further support the clinical relevance of IL-22 in patient stratification. Although IL-22-targeted therapies remain investigational, this pathway represents a promising biomarker and potential therapeutic target for chronic AD.

### 3.5. Emerging Role of Th9 Cells and IL-9

Although Th2 cells remain the dominant adaptive immune population in AD, increasing evidence suggests that Th9 cells also contribute to disease pathogenesis through IL-9 production. Th9 lymphocytes are characterized by the production of IL-9, a pleiotropic cytokine that enhances mast cell activation, promotes eosinophilic inflammation, and amplifies type 2 immune responses. Elevated IL-9 expression has been detected in lesional skin and peripheral blood of patients with AD, where it correlates with disease severity and chronic inflammation. Interactions between Th9- and Th2-driven pathways further increase the complexity of cytokine networks in AD and suggest that the IL-9 axis may serve as an additional biomarker and potential therapeutic target [13,20].

### 3.6. OX40/OX40L Signaling and T-Cell Activation

The OX40/OX40L signaling pathway has recently emerged as another important immunological target in AD [21,22,23]. The OX40/OX40L signaling pathway has recently emerged as an important regulator of sustained T-cell activation and chronic inflammation in AD [21,22,23]. Increased OX40/OX40L expression has been identified in lesional skin and is associated with persistent Th2 polarization, highlighting this pathway as a potential biomarker and therapeutic target. Elevated OX40/OX40L expression has been identified in AD skin lesions and appears associated with persistent Th2 polarization.

Because OX40 signaling influences multiple downstream cytokine pathways, including IL-4-, IL-13-, and IL-31-mediated responses, it represents an attractive therapeutic target in AD. Early clinical studies have shown encouraging reductions in inflammatory biomarkers and disease severity, whereas the clinical development of OX40-targeted therapies is discussed further in Section 6 [21,22,23].

### 3.7. Cytokine Networks, Biomarkers, and Precision Medicine

Collectively, the cytokine pathways discussed above illustrate that AD is driven by interconnected inflammatory networks rather than isolated immune mediators. Characterization of these networks provides a biological framework for immunological profiling, biomarker development, and individualized therapeutic decision-making [3,5].

**Figure 1:**
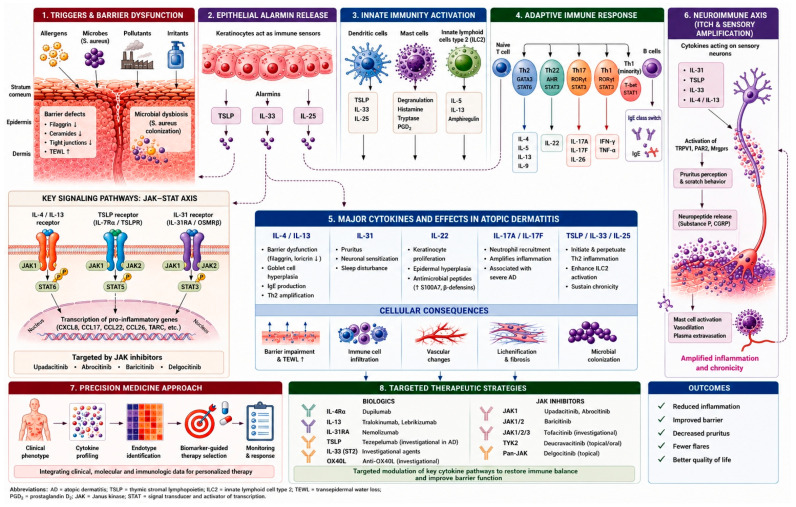
Mechanistic immunopathogenic pathways and precision-medicine targets in atopic dermatitis (Figure created in Canva). Environmental triggers, including allergens, microbial antigens, pollutants, and irritants, initiate epidermal barrier disruption characterized by filaggrin deficiency, altered lipid composition, impaired tight junction integrity, and increased transepidermal water loss (TEWL). Barrier dysfunction facilitates enhanced penetration of external antigens and promotes keratinocyte activation. Activated keratinocytes function as immune sensors and release epithelial-derived alarmins, including thymic stromal lymphopoietin (TSLP), interleukin (IL)-25, and IL-33, which initiate and amplify type 2 immune responses by activating dendritic cells, mast cells, basophils, and type 2 innate lymphoid cells (ILC2s). Adaptive immune activation is predominantly mediated by T helper 2 (Th2) cells producing IL-4, IL-5, and IL-13, leading to immunoglobulin E (IgE) class switching, eosinophilic inflammation, suppression of epidermal differentiation proteins, and amplification of barrier dysfunction. Additional inflammatory pathways involving Th22-, Th17-, and Th1-associated cytokines contribute to disease heterogeneity and chronic inflammation. IL-22 promotes keratinocyte proliferation, epidermal hyperplasia, and lichenification, whereas IL-17A/F enhances neutrophilic inflammation and inflammatory amplification. Interferon-γ (IFN-γ) produced by Th1 cells contributes to persistent immune activation. Neuroimmune interactions represent a major pathogenic component of atopic dermatitis. Cytokines such as IL-31, TSLP, IL-33, and IL-4/IL-13 directly stimulate sensory neurons through activation of itch-associated signaling pathways, including TRPV1, PAR2, and Mas-related G protein-coupled receptors (Mrgprs). IL-31-mediated neuronal sensitization promotes chronic pruritus and perpetuates the itch-scratch cycle, leading to further keratinocyte injury, mast cell activation, neurogenic inflammation, and disease chronicity. Central intracellular signaling pathways involved in atopic dermatitis include Janus kinase-signal transducer and activator of transcription (JAK-STAT) cascades activated downstream of IL-4/IL-13, TSLP, and IL-31 receptor signaling. Activation of these pathways induces transcription of pro-inflammatory genes, including CXCL8, CCL17, CCL22, CCL26, and thymus and activation-regulated chemokine (TARC). The lower panels illustrate the integration of cytokine profiling and immunological endotyping into precision medicine approaches. Identification of dominant inflammatory pathways may facilitate biomarker-guided therapeutic selection and individualized treatment strategies. Current targeted therapies include biologic agents targeting IL-4Rα, IL-13, IL-31 receptor α, TSLP, and OX40/OX40L signaling, as well as JAK inhibitors that suppress multiple cytokine-mediated inflammatory pathways. Collectively, these mechanisms highlight the transition of atopic dermatitis from a traditionally barrier-centered disease toward a highly heterogeneous systemic inflammatory disorder amenable to precision medicine interventions. Solid black arrows represent the direction of the mechanistic pathway linking sequential events.

The integrated interactions among epidermal barrier dysfunction, epithelial alarmins, adaptive and innate immune pathways, and neuroimmune signaling are schematically summarized in Figure 1, highlighting their contributions to disease pathogenesis and implications for targeted therapeutic strategies.

The increasing recognition of atopic dermatitis as a highly heterogeneous inflammatory disorder has highlighted the central role of complex cytokine interactions in shaping disease pathogenesis, chronicity, neuroimmune activation, epidermal remodeling, and therapeutic responsiveness. While classical type 2 cytokines remain dominant drivers of allergic inflammation, emerging mediators such as IL-31, IL-33, IL-22, and epithelial-derived alarmins have further expanded the current understanding of AD immunobiology and contributed to the identification of distinct inflammatory endotypes. To provide a comparative overview of the principal cytokines implicated in AD, Table 1 summarizes the major classical and emerging cytokines involved in AD pathogenesis, including their cellular origin, immunological pathways, biological functions, clinical significance, and corresponding targeted therapeutic strategies.

## 4. Biomarkers in Atopic Dermatitis: Toward Precision Medicine and Personalized Therapeutic Stratification

Building upon the cytokine networks discussed in the previous section, biomarkers have become central to improving disease stratification, therapeutic monitoring, and individualized management in AD [3,5]. Although clinical severity scores remain essential in routine practice, molecular and immunological biomarkers provide complementary information regarding disease activity and therapeutic responsiveness.

Biomarkers in AD encompass a broad spectrum of clinical, serological, cellular, transcriptomic, and cytokine-associated parameters reflecting different aspects of disease pathophysiology, including type 2 inflammation, epidermal barrier dysfunction, neuroimmune activation, and chronic tissue remodeling [3,16]. However, despite major advances in biomarker research, no single biomarker has yet demonstrated sufficient sensitivity and specificity to comprehensively capture the complexity of AD.

Current biomarker research has focused primarily on cytokine- and inflammation-related markers, including TARC/CCL17, periostin, eosinophil counts, serum IgE, IL-13, IL-22, and IL-31 [3]. These biomarkers are increasingly investigated as potential tools for disease stratification and treatment monitoring [3]. Nevertheless, the lack of standardized biomarker panels currently limits routine clinical implementation.

### 4.1. Clinical Biomarkers and Disease Severity Assessment

Clinical scoring systems remain the cornerstone of AD severity evaluation and continue to represent essential tools in both clinical practice and research settings [24,25]. Among the most widely used instruments are the Scoring Atopic Dermatitis (SCORAD) index, the Eczema Area and Severity Index (EASI), the Investigator’s Global Assessment (IGA), and the Dermatology Life Quality Index (DLQI). SCORAD integrates objective signs of inflammation with subjective symptoms such as pruritus and sleep disturbance, thereby providing a multidimensional assessment of disease burden [26,27]. In contrast, EASI primarily assesses objective inflammatory skin findings and is widely used in clinical trials evaluating biologic agents and JAKi [7,28,29].

Although clinical scoring systems remain indispensable for assessing disease severity and treatment response, they do not fully capture the underlying immunological heterogeneity of AD [3]. Consequently, patients with similar EASI or SCORAD scores may exhibit distinct cytokine signatures and therapeutic responses, highlighting the need for complementary molecular biomarkers [26,27].

Accordingly, integrating clinical assessment with molecular biomarkers may improve patient stratification and therapeutic decision-making, as discussed in the following sections [5].

### 4.2. Serum Biomarkers and Systemic Immune Activation

Several circulating biomarkers have been investigated as indicators of systemic inflammatory activity in AD [3]. Among the most extensively studied are total IgE, peripheral eosinophil counts, lactate dehydrogenase (LDH), thymus and activation-regulated chemokine (TARC/CCL17), and periostin.

Elevated serum IgE levels are a classical hallmark of extrinsic AD and reflect enhanced type 2 immune activation [1,16]. However, total IgE exhibits substantial interindividual variability and does not consistently correlate with disease severity, limiting its utility as a standalone biomarker [3].

Peripheral eosinophilia similarly reflects systemic type 2 inflammation and is frequently associated with severe disease phenotypes, increased pruritus intensity, and higher rates of atopic comorbidities [16]. Nevertheless, eosinophil counts may fluctuate considerably over time and may be influenced by concomitant allergic disorders.

Among currently available biomarkers, TARC/CCL17 has emerged as one of the most promising indicators of AD disease activity [30,31]. TARC is a chemokine involved in the recruitment of CCR4-positive Th2 lymphocytes into inflamed skin and has been shown to correlate strongly with SCORAD and EASI scores [32,33]. TARC levels also decrease following effective biologic therapy, suggesting their potential utility for treatment monitoring [3].

Periostin, an extracellular matrix protein induced predominantly by IL-13, has also gained attention as a biomarker associated with chronic inflammation, fibrosis, and tissue remodeling [34,35,36,37]. Elevated periostin concentrations have been linked to severe AD phenotypes and persistent disease activity.

Collectively, these findings indicate that serum biomarkers may provide valuable information regarding systemic immune activation, although their clinical applicability remains limited by insufficient specificity when used individually.

### 4.3. Cytokine-Based Biomarkers and Immunological Endotyping

Building upon the cytokine pathways discussed in Section 3, cytokine profiling has emerged as a valuable approach for biomarker development and immunological endotyping in AD [3,5]. Distinct cytokine signatures may support disease stratification and prediction of therapeutic response.

As discussed in Section 3.1, IL-13 is one of the most informative cytokine biomarkers in AD. Elevated IL-13 expression correlates with disease severity, while reductions following IL-4/IL-13-targeted therapy support its utility for treatment monitoring [12].

Similarly, IL-31 represents a biomarker of neuroimmune activation, with serum concentrations closely associated with pruritus severity, sleep disturbance, and disease activity (see Section 3.3) [6,10].

IL-22 has also been proposed as a biomarker of chronic epidermal remodeling and lichenification, particularly in longstanding AD (see Section 3.4) [13].

Epithelial alarmins, particularly TSLP and IL-33, are increasingly investigated as biomarkers of epithelial immune activation and early inflammatory responses (see Section 3.2) [6].

Importantly, cytokine expression profiles appear to vary according to age, ethnicity, disease chronicity, and treatment exposure [14,18,19]. Pediatric AD phenotypes frequently demonstrate stronger Th17/Th22 activation, whereas adult chronic disease is more strongly associated with IL-13- and IL-31-mediated pathways [4].

Collectively, these findings support integrating cytokine profiling into biomarker-guided patient stratification and therapeutic decision-making in AD.

### 4.4. Transcriptomic and Molecular Biomarkers

Beyond the serum biomarkers discussed above, transcriptomic technologies have substantially expanded understanding of molecular heterogeneity in AD [5]. High-throughput RNA sequencing and lesional skin transcriptomics have revealed distinct inflammatory signatures associated with disease severity, ethnicity, treatment response, and chronicity.

Transcriptomic analyses have demonstrated that lesional AD skin exhibits increased expression of genes associated with type 2 inflammation, epidermal hyperplasia, chemokine signaling, and neuroimmune activation [38,39,40,41]. Genes including CCL17, CCL22, IL13, IL31, S100A proteins, and periostin-related pathways are frequently upregulated in severe disease phenotypes.

Transcriptomic biomarkers may also predict therapeutic responsiveness. For example, IL-13-driven molecular signatures have been associated with improved responses to IL-4/IL-13-targeted therapies, whereas broader inflammatory profiles may identify patients more likely to benefit from JAK inhibitors (see Section 6) [5].

Despite these promising findings, transcriptomic profiling remains limited primarily to research settings due to high costs, technical complexity, and a lack of standardized methodologies.

### 4.5. Biomarkers and Therapeutic Response Prediction

One of the most clinically relevant applications of biomarkers in AD is predicting therapeutic response and optimizing individualized treatment strategies. The expanding therapeutic landscape, discussed in Section 6, has further highlighted the need for biomarkers that identify patients most likely to benefit from specific targeted therapies [5,28,29].

As discussed in Section 3 and Section 4.3, distinct cytokine signatures may predict differential therapeutic responses. Patients with dominant type 2 inflammatory profiles generally respond more favorably to IL-4/IL-13-targeted biologics, whereas broader inflammatory signatures may identify individuals more likely to benefit from JAK inhibitors [28,29].

Similarly, elevated IL-31 levels may identify patients more likely to benefit from IL-31-targeted therapy, including nemolizumab, as discussed in Section 3.3 and Section 6 [15].

Biomarkers may additionally facilitate monitoring of therapeutic efficacy and early identification of treatment resistance [3]. Dynamic changes in TARC, IL-13, eosinophil counts, and transcriptomic signatures have all demonstrated potential utility in longitudinal disease monitoring.

Nevertheless, substantial challenges remain regarding standardization, reproducibility, accessibility, and cost-effectiveness before biomarker-guided strategies can be routinely implemented in clinical practice. Large prospective studies are still required to validate these approaches.

### 4.6. Future Perspectives and Challenges in Biomarker Integration

Future advances in biomarker research will likely be driven by the integration of molecular profiling, multi-omics technologies, and artificial intelligence-assisted data analysis [2]. These approaches are expected to improve disease stratification and support more individualized therapeutic decision-making.

Future biomarker panels will likely require integrating multiple parameters rather than relying on isolated inflammatory mediators. Combined approaches incorporating cytokine profiles, serum biomarkers, transcriptomics, and clinical phenotyping may improve diagnostic precision and therapeutic prediction accuracy.

However, several challenges continue to limit routine clinical implementation, including the need for standardized laboratory methodologies, validation across diverse populations, reduced costs, and harmonized biomarker assessment strategies.

Despite these limitations, ongoing advances in biomarker discovery continue to redefine the molecular characterization of AD and are expected to improve future therapeutic personalization.

The principal biomarkers associated with disease activity, immunological profiling, and therapeutic response are summarized in Table 2.

## 5. Precision Medicine and Therapeutic Innovation in Atopic Dermatitis

Advances in molecular immunology have fundamentally transformed the therapeutic management of AD over the past decade [5,7]. Historically, treatment strategies for moderate-to-severe AD relied predominantly on nonspecific immunosuppressive agents, including systemic corticosteroids, cyclosporine, methotrexate, azathioprine, and mycophenolate mofetil. Although these therapies may reduce inflammatory activity, their long-term use is frequently limited by toxicity, inconsistent efficacy, and lack of selectivity [28,29].

Building upon the immunological mechanisms discussed in the previous sections, targeted biologic therapies and small-molecule inhibitors have enabled mechanism-based treatment of AD by selectively modulating key inflammatory pathways [5].

Current therapeutic strategies extend beyond broad immunosuppression to target specific molecular pathways implicated in barrier dysfunction, chronic pruritus, neuroimmune activation, and tissue remodeling. The following sections summarize the principal targeted therapies and their clinical applications.

### 5.1. Biologic Therapies Targeting Type 2 Inflammation

The development of biologic agents targeting IL-4 and IL-13 signaling pathways represents a major milestone in AD management and has validated type 2 inflammation as the dominant pathogenic axis in moderate-to-severe disease [7,8].

Dupilumab, a fully human monoclonal antibody targeting the IL-4 receptor α (IL-4Rα) subunit, inhibits signaling by both IL-4 and IL-13 [42,43,44]. Clinical trials have demonstrated substantial improvements in EASI scores, pruritus severity, sleep quality, and quality of life following dupilumab therapy [42,43,44]. Beyond its clinical efficacy, dupilumab also reduces biomarkers of epidermal barrier dysfunction and systemic type 2 inflammation, supporting its disease-modifying potential.

Long-term extension studies have confirmed sustained efficacy and favorable safety profiles associated with dupilumab treatment [45,46,47]. Nevertheless, conjunctivitis, facial erythema, and partial treatment responses remain clinically relevant limitations in certain patient populations.

Selective IL-13 inhibition has emerged as another important therapeutic strategy in AD. Tralokinumab [48,49,50,51,52,53,54,55,56] and lebrikizumab [57,58,59,60,61,62,63,64,65,66,67,68] selectively neutralize IL-13 and have demonstrated significant efficacy in reducing chronic inflammation, pruritus, and epidermal remodeling. These results further support selective IL-13 inhibition as an effective therapeutic strategy for patients with moderate-to-severe AD.

The identification of biomarkers associated with favorable responses to IL-4/IL-13 blockade is discussed in Section 4.5. Collectively, current evidence supports the use of immunological profiling to optimize patient selection for these therapies [3,5].

### 5.2. JAK Inhibitors and Broad Cytokine Modulation

JAKi represent another major therapeutic innovation in AD and differ fundamentally from biologic therapies in that they simultaneously inhibit intracellular signaling downstream of multiple cytokine receptors [7,28,29].

As discussed in Section 3, multiple cytokines implicated in AD signal through JAK-STAT pathways, making JAK inhibition an effective strategy for simultaneously modulating several inflammatory cascades [11].

Upadacitinib [65,66,67,68,69] and abrocitinib [70,71,72,73,74], both selective JAK1 inhibitors, have demonstrated rapid and substantial reductions in disease severity and pruritus intensity. Clinical improvement often occurs within days to weeks of treatment initiation, particularly in itch reduction, underscoring the importance of JAK-dependent neuroimmune signaling pathways.

Baricitinib, a JAK1/JAK2 inhibitor, has also shown efficacy in moderate-to-severe AD, although responses may be somewhat less pronounced compared with highly selective JAK1 inhibitors [75,76,77,78,79].

The broad therapeutic activity of JAK inhibitors may be particularly advantageous in patients with mixed inflammatory profiles or incomplete responses to biologic therapy [28,29]. However, safety considerations remain highly relevant. Potential adverse effects include infections, acneiform eruptions, laboratory abnormalities, thromboembolic events, and cardiovascular risk concerns [70,71,72,73,74]. Consequently, careful patient selection and long-term pharmacovigilance remain essential.

### 5.3. Targeting Neuroimmune Pathways and Pruritus

Pruritus remains one of the most debilitating symptoms in AD and a major therapeutic challenge. Traditional anti-inflammatory therapies often fail to achieve adequate itch control, highlighting the need for direct modulation of neuroimmune pathways [6,10].

As discussed in Section 3.3, IL-31 represents the principal cytokine involved in neuroimmune signaling and chronic pruritus. Therapeutic blockade of the IL-31 receptor with nemolizumab has demonstrated rapid reductions in itch severity, sleep disturbance, and scratching behavior, with antipruritic effects often preceding improvements in skin inflammation [15]. The efficacy and safety profile of nemolizumab, including cutaneous adverse events, are discussed in Section 6.

Additional neuroimmune therapeutic strategies are under investigation, particularly those targeting epithelial alarmins such as TSLP and IL-33 (see Section 3.2). Modulation of these pathways may provide both anti-inflammatory and antipruritic benefits [6,10].

Collectively, these advances highlight the growing importance of neuroimmune-targeted therapies as complementary components of modern AD management.

### 5.4. Emerging Therapeutic Targets Beyond Type 2 Immunity

As discussed in Section 3, several inflammatory pathways beyond the classical IL-4/IL-13 axis have emerged as potential therapeutic targets in AD [1,4]. Current therapeutic development increasingly focuses on these alternative immune pathways to address disease heterogeneity and variable treatment responses.

As outlined in Section 3.6, OX40/OX40L signaling is a promising therapeutic target due to its role in sustaining chronic T-cell activation. Early clinical studies of anti-OX40 monoclonal antibodies have demonstrated encouraging reductions in inflammatory biomarkers and disease severity [21,22,23].

Therapies targeting epithelial alarmins, particularly TSLP and IL-33, are also undergoing clinical evaluation (see Section 3.2). Because these cytokines act upstream in the inflammatory cascade, their inhibition may modulate multiple downstream immune pathways [7].

Additional investigational approaches involve modulation of IL-22 signaling, Th17-associated pathways, and microbiome-associated immune regulation [13]. These strategies may expand therapeutic options for patients with more complex inflammatory profiles.

Collectively, these emerging therapeutic strategies illustrate the continuing evolution of mechanism-based treatment in AD and are expected to further expand individualized therapeutic options.

### 5.5. Biomarker-Guided Therapy and Precision Medicine Algorithms

As discussed in Section 4, the integration of biomarkers into therapeutic decision-making represents a central objective of personalized AD management [5]. Biomarker-guided strategies aim to improve the prediction of therapeutic response and optimize treatment selection.

Examples of biomarker-guided therapeutic selection are presented in Section 4.5. Briefly, dominant type 2 inflammatory profiles are generally associated with better responses to IL-4/IL-13-targeted therapies, whereas broader inflammatory signatures may favor treatment with JAK inhibitors. Likewise, elevated IL-31 expression may identify patients more likely to benefit from neuroimmune-targeted therapies such as nemolizumab [5,15,28,29].

Emerging transcriptomic studies additionally suggest that molecular profiling may predict treatment responsiveness prior to therapeutic initiation [38,39,40,41]. Such approaches could ultimately reduce therapeutic failure, minimize unnecessary immunosuppression, and improve long-term disease control.

Artificial intelligence and machine learning may facilitate the integration of clinical, molecular, transcriptomic, and biomarker datasets, thereby improving patient stratification and therapeutic decision-making in AD [80,81,82,83].

Despite these advances, routine implementation of biomarker-guided treatment algorithms will require standardized biomarker panels, broader validation across diverse populations, improved accessibility, and cost-effective molecular profiling technologies.

### 5.6. Future Perspectives in Therapeutic Innovation

Future therapeutic advances in AD are expected to focus on integrating targeted interventions with biomarker-guided treatment selection [5]. Combination therapies directed at multiple inflammatory pathways may be particularly beneficial for patients with severe or treatment-resistant disease.

Advances in single-cell transcriptomics, spatial immunology, and multi-omics integration continue to improve understanding of disease heterogeneity and may ultimately facilitate real-time monitoring of inflammatory activity and therapeutic response [84,85,86,87].

Furthermore, early intervention strategies targeting epithelial alarmins and neuroimmune pathways may potentially alter disease progression and prevent chronic inflammatory remodeling. Such approaches could shift AD management from reactive symptom control toward proactive disease modification.

Collectively, these advances are expected to further expand mechanism-based therapeutic options and improve long-term management of AD.

**Figure 2:**
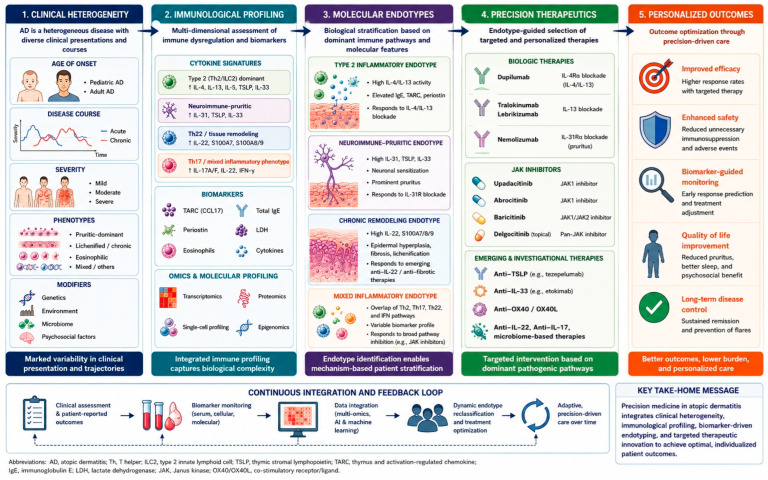
Conceptual framework of precision medicine in atopic dermatitis: from immunopathogenesis and biomarker profiling to endotype-guided therapeutic innovation and personalized outcomes (Figure created in Canva). This figure illustrates an integrated precision medicine model for atopic dermatitis (AD), emphasizing the transition from conventional phenotype-based management toward biomarker-driven and endotype-guided therapeutic strategies. (1) The framework begins with the recognition of substantial clinical heterogeneity in AD, including variations in age of onset, disease course, severity, inflammatory phenotype, and environmental or genetic modifiers. These factors contribute to marked interindividual variability in disease presentation and progression. (2) *The second panel* highlights immunological profiling as a central component of precision medicine in AD. Cytokine signatures associated with type 2 inflammation, neuroimmune activation, tissue remodeling, and mixed inflammatory responses are integrated with serum biomarkers, including thymus and activation-regulated chemokine (TARC/CCL17), immunoglobulin E (IgE), eosinophils, lactate dehydrogenase (LDH), and periostin. Advanced molecular approaches, including transcriptomics, proteomics, epigenomics, and single-cell profiling, further characterize disease-specific immune dysregulation and the underlying biological complexity. (3) *The third panel* demonstrates the identification of distinct molecular endotypes based on dominant immunological pathways. The type 2 inflammatory endotype is characterized by increased IL-4/IL-13 activity, elevated IgE, TARC, and periostin expression, and favorable responses to IL-4/IL-13 blockade. The neuroimmune-pruritic endotype is associated with enhanced signaling by IL-31, TSLP, and IL-33, neuronal sensitization, and severe chronic pruritus. The chronic remodeling endotype is characterized by IL-22-driven epidermal hyperplasia, fibrosis, and lichenification, whereas the mixed inflammatory endotype involves overlapping Th2-, Th17-, Th22-, and interferon-associated pathways with broader inflammatory activation and variable therapeutic responsiveness. (4) *The fourth panel* illustrates precision therapeutics targeting specific pathogenic pathways. Biologic agents, including dupilumab, tralokinumab, lebrikizumab, and nemolizumab, selectively inhibit major cytokine axes involved in AD immunopathogenesis. Small-molecule therapies, such as JAK inhibitors, simultaneously suppress multiple downstream inflammatory signaling pathways. Emerging and investigational therapies targeting TSLP, IL-33, OX40/OX40L, IL-22, IL-17, and microbiome-associated pathways reflect the ongoing expansion of mechanism-based therapeutic approaches. (5) *The final panel* summarizes the principal objectives of precision medicine in AD, including improved therapeutic efficacy, enhanced safety through reduction of unnecessary immunosuppression, biomarker-guided monitoring, improved quality of life, and sustained long-term disease control. *The lower section* emphasizes the importance of integrating clinical assessment, biomarker monitoring, multi-omics analysis, and dynamic endotype reclassification to optimize adaptive, individualized therapeutic strategies over time. Upward arrows (↑) indicate increased expression, activation, or pathological enhancement.

Figure 2 summarizes the relationships among immunopathogenesis, biomarker profiling, molecular endotyping, and targeted therapeutic strategies discussed throughout this review.

## 6. Therapeutic Innovations in Atopic Dermatitis

As outlined in Section 5, advances in molecular immunology have fundamentally transformed the therapeutic management of AD [5,7,8,88,89]. Historically, treatment strategies for moderate-to-severe AD relied predominantly on broad immunosuppressive agents such as systemic corticosteroids, cyclosporine, methotrexate, azathioprine, and mycophenolate mofetil. Although these therapies may reduce inflammatory activity, their long-term use is frequently limited by toxicity, variable efficacy, and lack of immunological selectivity [28,29].

Current targeted therapies selectively modulate key cytokine pathways involved in AD pathogenesis, including type 2 inflammation, neuroimmune activation, epithelial alarmin signaling, and chronic inflammatory amplification [7,90,91,92]. The principal therapeutic classes and their mechanisms are summarized in the following sections.

Beyond controlling cutaneous inflammation, current therapeutic strategies aim to restore epidermal barrier function, reduce chronic pruritus, and achieve sustained disease control through mechanism-based interventions. The following sections review the available biologic therapies, JAK inhibitors, and emerging targeted agents.

### 6.1. Biologic Therapies Targeting IL-4 and IL-13 Signaling

The introduction of biologic therapies targeting IL-4 and IL-13 pathways represented a major breakthrough in AD treatment and validated type 2 inflammation as the dominant pathogenic axis in moderate-to-severe disease [42,43,44,45,46,47].

Dupilumab is a fully human monoclonal antibody directed against the interleukin-4 receptor alpha (IL-4Rα) subunit, thereby inhibiting signaling mediated by both IL-4 and IL-13 [42,43,44]. By blocking this shared receptor pathway, dupilumab suppresses Th2-mediated immune responses, improves epidermal barrier function, reduces eosinophilic inflammation, and significantly alleviates. Large phase III clinical trials and long-term extension studies have demonstrated substantial improvements in EASI and SCORAD scores, sleep quality, patient-reported outcomes, and overall favorable safety [42,43,44,45,46,47]. As the first biologic approved for moderate-to-severe AD, dupilumab has fundamentally transformed disease management and provided clinical validation of the central role of IL-4/IL-13 signaling in AD pathogenesis.

Selective IL-13 inhibition has subsequently emerged as another important therapeutic strategy. Tralokinumab is a fully human IgG4 monoclonal antibody that selectively neutralizes IL-13, preventing its interaction with both IL-13Rα1 and IL-13Rα2 receptors. Through specific inhibition of IL-13-dependent pathways, tralokinumab reduces keratinocyte activation, epidermal barrier impairment, and chronic skin inflammation. Clinical studies have demonstrated significant improvements in disease severity and pruritus, further supporting IL-13 as a key therapeutic target in AD [50,51,54].

Lebrikizumab is a high-affinity monoclonal antibody that binds soluble IL-13 and prevents the formation of the IL-13Rα1/IL-4Rα signaling complex while preserving its interaction with the decoy receptor, IL-13Rα2. This selective mechanism attenuates IL-13-driven inflammation, tissue remodeling, and skin barrier dysfunction. Clinical trials have demonstrated substantial improvements in EASI scores, pruritus, and quality of life, supporting the clinical relevance of selective IL-13 inhibition in AD [50,61,64].

Collectively, dupilumab, tralokinumab, and lebrikizumab have established IL-4/IL-13 blockade as the cornerstone of targeted therapy for moderate-to-severe AD. Variability in treatment response further supports the role of biomarker-guided patient selection, as discussed in Section 4 [4,7,28,61].

### 6.2. JAK Inhibitors and Intracellular Cytokine Signaling Modulation

JAKi represent another major therapeutic innovation in AD and differ fundamentally from biologic agents in that they simultaneously inhibit intracellular signaling downstream of multiple cytokine receptors [48,49,50,51,52,53,54,55,56,57,58,59,60,61,62,93,94,95].

As discussed in Section 3 and Section 5.2, multiple cytokines implicated in AD signal through JAK-STAT pathways, making JAK inhibition an effective strategy for simultaneously modulating several inflammatory cascades [11].

Upadacitinib and abrocitinib, both selective JAK1 inhibitors, have demonstrated rapid and substantial improvements in inflammatory skin lesions and pruritus severity [65,66,67,68,69,70,71,72,73,74]. Clinical improvement often occurs within days of treatment initiation, particularly in itch reduction, underscoring the importance of JAK-dependent neuroimmune signaling pathways in AD.

Baricitinib, a JAK1/JAK2 inhibitor, has also demonstrated efficacy in moderate-to-severe disease, although clinical responses may be somewhat less pronounced than those observed with highly selective JAK1 inhibitors [75,76,77,78,79].

The broad immunomodulatory activity of JAK inhibitors may be particularly advantageous in patients with mixed inflammatory profiles or incomplete responses to biologic therapy [28,29].

Nevertheless, safety considerations remain highly relevant in the context of JAKi. Potential adverse effects include infections, acneiform eruptions, laboratory abnormalities, thromboembolic events, and cardiovascular risk concerns [70,71,72,73,74]. Consequently, careful patient selection and long-term pharmacovigilance remain essential components of therapeutic management.

### 6.3. Neuroimmune-Targeted Therapies and Pruritus Modulation

Pruritus represents one of the most debilitating clinical manifestations of AD and remains a major therapeutic challenge [6,10]. Conventional anti-inflammatory therapies often fail to fully control chronic itch, underscoring the importance of direct neuroimmune modulation.

As discussed in Section 3.3, IL-31 is a central mediator of neuroimmune signaling and chronic pruritus in AD [6].

Nemolizumab, a monoclonal antibody targeting the IL-31 receptor α subunit, has demonstrated rapid and significant antipruritic effects in moderate-to-severe AD. Clinical trials showed marked reductions in itch severity, sleep disturbance, and scratching behavior, often preceding visible improvement in inflammatory skin lesions [15,96,97,98,99]. These findings establish IL-31 blockade as an effective therapeutic strategy for controlling chronic pruritus in AD.

Although nemolizumab has demonstrated substantial efficacy in reducing pruritus and improving disease control, increasing attention has been paid to cutaneous adverse events during treatment. Reported reactions include eczema-like eruptions, paradoxical worsening of AD lesions, psoriasiform dermatitis, dyshidrotic eczema, nummular eczema, alopecia, and, in rare cases, bullous pemphigoid. Most events occur during the first months of therapy and are generally manageable with topical treatment without requiring drug discontinuation. The underlying mechanisms remain incompletely understood but may involve alterations in the balance of the cytokine network following IL-31 blockade, resulting in relative shifts toward Th2-, Th17-, or other inflammatory pathways. Importantly, the heterogeneity of these reactions highlights the complexity of AD immunopathogenesis and suggests that individual immunological endotypes may influence both therapeutic response and susceptibility to adverse events. Consequently, characterization of nemolizumab-associated cutaneous reactions may improve patient stratification and guide future biomarker-based therapeutic approaches [15,17,100,101,102].

Additional neuroimmune therapeutic strategies targeting epithelial alarmins, particularly TSLP and IL-33, are currently under investigation (see Section 3.2). These approaches may provide complementary anti-inflammatory and antipruritic benefits [6].

### 6.4. Emerging Therapeutic Targets Beyond Classical Type 2 Inflammation

As discussed in Section 3.2, Section 3.3, Section 3.4, Section 3.5 and Section 3.6, several inflammatory pathways beyond the IL-4/IL-13 axis have emerged as potential therapeutic targets in AD [1,4]. Ongoing drug development increasingly focuses on these alternative pathways to expand treatment options for patients with heterogeneous disease profiles.

OX40/OX40L-targeted therapies, discussed in Section 3.6, aim to reduce persistent T-cell activation and chronic inflammation. Early clinical studies suggest that inhibition of this pathway may modulate multiple downstream inflammatory responses [16,21,22,23].

Therapies targeting epithelial alarmins, particularly TSLP and IL-33, are also under active clinical investigation (see Section 3.2). By acting upstream in the inflammatory cascades, these agents may provide broader immunomodulatory effects [7].

IL-22-targeted therapies are being investigated for patients with chronic AD characterized by epidermal remodeling and lichenification, consistent with the pathogenic role of IL-22 discussed in Section 3.4 [13].

Additional investigational strategies include modulation of Th17-associated pathways, restoration of epidermal barrier integrity, microbiome-directed therapy, and epigenetic regulation [13,103,104]. Together, these approaches broaden the therapeutic landscape and may further improve individualized management of AD.

### 6.5. Precision Therapeutics and Endotype-Guided Treatment Strategies

As discussed in Section 4, advances in biomarker research have provided the foundation for endotype-guided therapeutic strategies in AD [5,84,85,86,87]. The goal of these approaches is to improve treatment selection by integrating clinical and molecular information.

Examples of biomarker-guided therapeutic stratification are presented in Section 4.5. In general, dominant type 2 inflammatory profiles are associated with favorable responses to IL-4/IL-13-targeted biologics, whereas broader inflammatory signatures may identify patients more likely to benefit from JAK inhibitors [3,5].

Likewise, elevated IL-31 expression may support selection of patients for neuroimmune-targeted therapies such as nemolizumab (see Section 6.3) [15].

The future integration of transcriptomic profiling, single-cell analysis, and artificial intelligence-assisted biomarker interpretation may further refine therapeutic stratification and facilitate highly individualized therapeutic algorithms [79,80,81,82,83,84,85,86].

Beyond improving therapeutic efficacy, endotype-guided treatment strategies aim to minimize unnecessary immunosuppression, reduce adverse effects, improve long-term adherence, and optimize patient quality of life.

### 6.6. Challenges and Future Perspectives in Therapeutic Innovation

Despite remarkable therapeutic progress, several important challenges remain in implementing precision therapeutics in routine clinical practice [5]. Significant overlap exists among inflammatory endotypes, and dynamic patterns of cytokine expression may complicate therapeutic prediction.

Additionally, substantial variability in treatment responsiveness persists even among patients with apparently similar immunological profiles. The lack of universally standardized biomarker panels currently limits precise therapeutic stratification.

Long-term safety monitoring also remains essential, particularly regarding chronic JAKi exposure and prolonged cytokine suppression [70,71,72,73,74]. Further studies are required to clarify optimal sequencing strategies, combination therapies, and the long-term disease-modifying potential of emerging agents.

Despite these challenges, ongoing therapeutic advances are expected to further improve personalized treatment strategies and expand the range of mechanism-based interventions available for patients with AD.

Table 3 summarizes the principal targeted therapies currently available or under clinical investigation for AD, including their molecular targets, mechanisms of action, principal clinical effects, and major therapeutic limitations.

## 7. Future Directions and Emerging Perspectives in Precision Medicine for Atopic Dermatitis

Building on the advances discussed in the preceding sections, future research in AD is expected to focus on refining precision medicine through improved biomarker discovery, molecular profiling, and individualized therapeutic strategies [84,85,86,87]. Although substantial progress has been achieved, several important scientific and clinical challenges remain unresolved.

Future research is expected to refine disease endotyping, integrate multi-omics technologies, and validate predictive biomarkers for routine clinical use [3,5]. Successful implementation will depend on translating molecular insights into practical therapeutic decision-making.

### 7.1. Expanding the Concept of Immunological Endotypes

Further refinement of immunological endotypes remains an important research priority because it may improve disease stratification and therapeutic selection [5]. As discussed throughout this review, patients with AD exhibit considerable variability in inflammatory pathways, emphasizing the need for clinically applicable molecular endotypes [3,14].

Future studies should clarify how individual inflammatory pathways influence disease progression and therapeutic responsiveness across different patient populations [4,6].

Furthermore, increasing evidence suggests that endotypes may evolve dynamically over time in response to age, environmental exposures, microbiome alterations, and therapeutic intervention [5]. Consequently, future precision medicine frameworks will likely require continuous reassessment of molecular profiles rather than static classification systems.

### 7.2. Multi-Omics Technologies and Systems Immunology

Rapid advances in multi-omics technologies are expected to provide a more comprehensive understanding of the molecular mechanisms underlying AD [84,85,86,87]. Integrated analysis of transcriptomics, proteomics, metabolomics, epigenomics, lipidomics, and single-cell sequencing may reveal novel pathogenic pathways and clinically relevant molecular signatures.

Single-cell RNA sequencing has already demonstrated remarkable potential for identifying disease-specific immune cell populations, cytokine-producing cell subsets, and spatial inflammatory microenvironments in lesional AD skin [105]. These technologies may facilitate the identification of previously unrecognized pathogenic pathways and novel therapeutic targets.

Spatial transcriptomics represents another rapidly evolving field with particular relevance for inflammatory skin diseases. By enabling simultaneous analysis of gene expression and tissue architecture, spatial profiling technologies may improve understanding of interactions between keratinocytes, immune cells, fibroblasts, endothelial cells, and peripheral sensory neurons within the AD microenvironment [106].

Integration of multi-omics datasets may ultimately improve the prediction of therapeutic response and support the development of more robust biomarker-guided treatment strategies.

### 7.3. Artificial Intelligence and Predictive Modeling

Artificial intelligence (AI) and machine learning technologies are expected to become increasingly important for integrating large-scale clinical, molecular, transcriptomic, imaging, and biomarker datasets in AD [80,81,82,83]. These technologies may support predictive models that improve diagnostic and treatment decision-making.

Machine learning models may identify complex biomarker combinations associated with treatment responsiveness, disease progression, or therapeutic resistance that are not detectable with conventional statistical approaches [107]. Such technologies could facilitate real-time treatment optimization and continuous monitoring of disease evolution. AI-assisted image analysis may additionally improve objective assessment of disease severity and longitudinal treatment monitoring. Integration of digital phenotyping with molecular biomarkers may further improve individualized treatment strategies. Nevertheless, substantial challenges remain regarding data standardization, algorithm validation, interpretability, patient privacy, and integration into routine clinical workflows [107]. Ethical considerations surrounding AI-assisted decision-making will also require careful evaluation.

### 7.4. Future Biomarkers and Noninvasive Monitoring Strategies

One of the major limitations of current biomarker implementation in AD involves the lack of standardized, reproducible, and clinically accessible molecular monitoring tools [3]. Current research, therefore, focuses on developing minimally invasive and noninvasive monitoring technologies suitable for routine clinical practice.

Tape-strip sampling has emerged as a particularly promising approach for evaluating epidermal cytokine expression and barrier-associated molecular signatures without requiring invasive skin biopsies [38,39,40,41]. Such techniques may facilitate serial monitoring of inflammatory activity and treatment response in both pediatric and adult patients.

Emerging monitoring approaches include wearable biosensors, sweat analysis, microbiome-derived biomarkers, and real-time inflammatory monitoring technologies [108,109,110,111]. Their integration into clinical practice could improve longitudinal disease assessment and therapeutic adjustment.

Future biomarker strategies will likely rely on composite signatures that integrate cytokine profiles, transcriptomic data, serum biomarkers, and clinical characteristics, thereby improving the prediction of disease activity and therapeutic response.

### 7.5. Emerging Therapeutic Strategies and Disease Modification

Future therapeutic strategies in AD are expected to move beyond symptomatic control toward sustained disease modification and restoration of long-term immune tolerance [28,29]. Several investigational approaches may further expand the available treatment options.

As discussed in Section 6.4, therapies targeting epithelial alarmins (TSLP and IL-33) and the OX40/OX40L pathway remain promising strategies for preventing early immune activation and sustaining long-term disease control [6,7,17,21,22,23].

Combination therapies targeting complementary inflammatory pathways may also become increasingly relevant in severe or treatment-resistant disease phenotypes. Targeting complementary pathogenic pathways may improve long-term outcomes, particularly in patients with complex or treatment-resistant disease.

Ultimately, future therapeutic strategies may aim not only to control inflammation but also to achieve sustained remission, prevent chronic tissue remodeling, and interrupt the atopic march.

### 7.6. Challenges in Implementing Precision Medicine in Clinical Practice

Despite remarkable scientific progress, several barriers continue to limit routine implementation of biomarker-guided therapeutic strategies in AD. One of the principal challenges involves the substantial overlap between inflammatory endotypes and the dynamic nature of cytokine expression profiles.

Additionally, many molecular profiling technologies remain expensive, technically complex, and largely restricted to research environments. Standardization of laboratory methodologies and validation across ethnically diverse populations remain essential priorities before widespread clinical implementation becomes feasible.

Another important challenge involves interpreting complex biomarker datasets in routine clinical practice. Integration of transcriptomics, cytokine profiling, and multi-omics data requires substantial bioinformatic infrastructure and interdisciplinary collaboration between dermatologists, immunologists, molecular biologists, and computational scientists.

Furthermore, disparities in healthcare access may significantly influence the global applicability of precision medicine strategies. Ensuring equitable access to advanced molecular diagnostics and targeted therapies will remain an important ethical and public health consideration in the future.

### 7.7. Future and Translational Perspective

The rapid expansion of knowledge regarding cytokine biology, molecular endotypes, and neuroimmune mechanisms has created new opportunities for precision medicine in AD. Future progress will likely depend on integrating immunological profiling, multi-omics technologies, digital health platforms, and artificial intelligence–assisted predictive models to identify individualized disease trajectories and therapeutic responses.

Despite these advances, several challenges remain, including biomarker standardization, validation of clinically relevant endotypes, cost-effective implementation of molecular diagnostics, and the development of accessible monitoring tools suitable for routine clinical practice. Addressing these challenges will require close collaboration among dermatologists, immunologists, bioinformaticians, and translational researchers.

Ultimately, a deeper understanding of disease heterogeneity may facilitate the transition from reactive symptom control toward proactive, mechanism-based, and highly individualized therapeutic strategies tailored to each patient’s unique immunological profile.

## 8. Conclusions

AD is increasingly recognized as a highly heterogeneous inflammatory disorder in which epidermal barrier dysfunction, immune dysregulation, epithelial-derived signaling, and neuroimmune interactions collectively contribute to disease pathogenesis and clinical variability. While IL-4 and IL-13 remain central drivers of type 2 inflammation, emerging mediators such as IL-31, IL-33, IL-22, TSLP, and OX40/OX40L signaling pathways further shape disease chronicity, pruritus, tissue remodeling, and immunological heterogeneity. The expanding characterization of cytokine networks has improved understanding of AD endotypes and has supported the identification of biomarkers with potential applications in disease stratification, treatment monitoring, and therapeutic selection. In parallel, the development of biologic agents and JAK inhibitors has transformed the therapeutic landscape and provided a practical framework for implementing precision medicine in clinical dermatology. The conceptual model proposed in this review integrates immunopathogenesis, biomarker-driven profiling, molecular endotypes, and targeted therapeutic innovation into a unified precision medicine framework. Continued refinement of immunological profiling strategies and biomarker-guided treatment algorithms may further improve individualized care and long-term outcomes for patients with AD.

## Figures and Tables

**Table 1:** Classical and emerging cytokines involved in the immunopathogenesis of atopic dermatitis (AD).

Cytokine	Main Cellular Source	Signaling Pathway	Major Biological Effects	Clinical Relevance	Targeted Therapy
IL-4	Th2 cells, ILC2s	JAK1/STAT6	IgE switching, Th2 polarization, barrier dysfunction	Acute inflammation	Dupilumab
IL-13	Th2 cells, ILC2s	JAK1/TYK2/STAT6	Epidermal remodeling, chronic inflammation	Disease severity	Tralokinumab, Lebrikizumab
IL-5	Th2 cells	JAK/STAT	Eosinophil activation and survival	Eosinophilic inflammation	Investigational
IL-31	Th2 cells	JAK1/STAT3	Pruritus, neuronal sensitization	Itch severity	Nemolizumab
IL-22	Th22 cells	STAT3	Keratinocyte proliferation, lichenification	Chronic lesions	Investigational
IL-17A/F	Th17 cells	ACT1/NF-κB	Neutrophilic inflammation	Specific endotypes	Investigational
IFN-γ	Th1 cells	JAK1/JAK2/STAT1	Chronic inflammation	Long-standing disease	None approved
TSLP	Keratinocytes	TSLPR/JAK-STAT	Th2 initiation, dendritic activation	Early inflammation	Tezepelumab (investigational)
IL-33	Keratinocytes	ST2/NF-κB/MAPK	Alarmin amplification, neuroinflammation	Severe AD, pruritus	Investigational
IL-25	Keratinocytes	IL-17RB/NF-κB	ILC2 activation	Early allergic inflammation	Investigational
OX40/OX40L	T cells/DCs	NF-κB	T-cell survival and cytokine amplification	Chronic inflammation	Anti-OX40 agents

This table summarizes the principal cytokines implicated in the pathogenesis of AD, including both classical type 2 inflammatory mediators and emerging cytokines associated with neuroimmune activation, epithelial-derived signaling, epidermal remodeling, and chronic inflammatory amplification. For each cytokine, the major cellular source, immunological pathway, biological effects, clinical relevance, and corresponding targeted therapeutic strategies are presented. Comparative evaluation of these cytokine pathways highlights the substantial immunological heterogeneity of AD and supports the development of biomarker-driven precision medicine approaches. AD, atopic dermatitis; IL, interleukin; ILC2s, type 2 innate lymphoid cells; IFN-γ, interferon gamma; TSLP, thymic stromal lymphopoietin; IgE, immunoglobulin E; OX40L, OX40 ligand.

**Table 2:** Potential biomarkers associated with disease severity and therapeutic response in atopic dermatitis.

Biomarker	Biological Source	Pathophysiological Role	Clinical Association	Potential Therapeutic Implications
Total IgE	B cells	Type 2 immune activation	Extrinsic AD, allergic sensitization	Predictive of Th2-dominant disease
Eosinophils	Peripheral blood	Eosinophilic inflammation	Severe disease phenotypes	Response to type 2-targeted therapies
TARC/CCL17	Dendritic cells, keratinocytes	Th2 cell recruitment	Correlates with SCORAD/EASI	Monitoring treatment response
Periostin	Fibroblasts, keratinocytes	Tissue remodeling, fibrosis	Chronic AD	Predictor of IL-13-driven inflammation
IL-13	Th2 cells	Chronic type 2 inflammation	Severe AD, chronic lesions	Response to IL-13 inhibitors
IL-31	Th2 cells	Neuroimmune pruritus signaling	Severe itch, sleep disturbance	Response to nemolizumab
IL-22	Th22 cells	Epidermal hyperplasia	Lichenified/chronic AD	Marker of remodeling phenotype
TSLP	Keratinocytes	Epithelial alarmin activation	Early inflammation	Candidate for upstream targeted therapy
IL-33	Keratinocytes	Amplification of Th2 inflammation	Severe inflammatory phenotype	Emerging therapeutic target
LDH	Serum biomarker	Tissue inflammation and turnover	Disease activity	General inflammatory marker
Transcriptomic signatures	Lesional skin	Molecular endotyping	Disease heterogeneity	Precision therapeutic stratification
JAK-STAT activation profile	Multiple immune cells	Cytokine signaling amplification	Broad inflammatory activation	Candidate for JAK inhibitor responsiveness

This table summarizes major serum, cytokine-associated, cellular, and molecular biomarkers currently investigated in atopic dermatitis. The presented biomarkers are associated with different aspects of disease pathophysiology, including type 2 inflammation, neuroimmune activation, epidermal remodeling, and systemic inflammatory activity. Their potential utility for disease stratification, immunological endotyping, prediction of therapeutic response, and biomarker-guided precision medicine approaches is also highlighted. AD, atopic dermatitis; IgE, immunoglobulin E; TARC/CCL17, thymus and activation-regulated chemokine/C-C motif chemokine ligand 17; IL, interleukin; TSLP, thymic stromal lymphopoietin; LDH, lactate dehydrogenase; JAK-STAT, Janus kinase-signal transducer and activator of transcription.

**Table 3:** Targeted therapies and molecular pathways in atopic dermatitis (AD).

Therapy	Molecular Target	Mechanism of Action	Main Clinical Effects	Limitations/Safety Concerns
Dupilumab	IL-4Rα	Blocks IL-4/IL-13 signaling	Reduces inflammation and pruritus	Conjunctivitis
Tralokinumab	IL-13	Selective IL-13 inhibition	Improves chronic lesions	Partial responders
Lebrikizumab	IL-13	IL-13 neutralization	Reduces EASI and itch	Long-term data limited
Nemolizumab	IL-31RA	Inhibits neuroimmune itch signaling	Rapid antipruritic effect	Injection-site reactions
Upadacitinib	JAK1	Broad cytokine signaling inhibition	Rapid disease control	Infection risk
Abrocitinib	JAK1	Suppresses multiple inflammatory pathways	Fast itch reduction	Thromboembolic warnings
Baricitinib	JAK1/JAK2	Multi-cytokine inhibition	Moderate efficacy	Laboratory abnormalities
Delgocitinib	Pan-JAK	Topical cytokine inhibition	Local inflammation control	Limited availability
Tezepelumab	TSLP	Alarmin inhibition	Experimental disease modulation	Investigational
Anti-OX40 agents	OX40/OX40L	T-cell co-stimulation blockade	Reduces chronic inflammation	Early clinical phase

Overview of currently approved and emerging targeted therapies for atopic dermatitis, including biologic agents and Janus kinase (JAK) inhibitors. The table summarizes the principal molecular targets, mechanisms of action, major clinical effects, and key safety considerations for each therapeutic class. These therapies illustrate the transition from broad immunosuppressive approaches toward mechanism-based and endotype-guided precision therapeutics in AD. AD, atopic dermatitis; IL, interleukin; IL-4Rα, interleukin-4 receptor alpha; IL-31RA, interleukin-31 receptor alpha; JAK, Janus kinase; TYK2, tyrosine kinase 2; EASI, Eczema Area and Severity Index.

## Data Availability

No new data were created or analyzed in this study. Data sharing is not applicable to this article.
